# Supplementary material for: Novel regulators of islet function identified from genetic variation in mouse islet Ca2+ oscillations
Source: eLife. 2023 Oct 3;12:RP88189. doi: 10.7554/eLife.88189 (PMC10547476; doi:10.7554/eLife.88189)
Supplement: MDAR checklist [file elife-88189-mdarchecklist1.docx]

**Materials Design Analysis Reporting (MDAR)**

**Checklist for Authors**

The MDAR framework establishes a minimum set of requirements in transparent reporting applicable to studies in the life sciences (see Statement of Task: doi:10.31222/osf.io/9sm4x.). The MDAR checklist is a tool for authors, editors, and others seeking to adopt the MDAR framework for transparent reporting in manuscripts and other outputs. Please refer to the MDAR Elaboration Document for additional context for the MDAR framework.

**For all that apply, please note where in the manuscript the required information is provided.**

**Materials:**

| **Newly created materials** | **indicate where provided: page no/section/legend)** | **n/a** |
| --- | --- | --- |
| The manuscript includes a dedicated "materials availability statement" providing transparent disclosure about availability of newly created materials including details on how materials can be accessed and describing any restrictions on access. | Calcium data resource link is on pages 27-29. There are no newly created reagents or physical materials. |  |
|  |  |  |
| **Antibodies** | **indicate where provided: page no/section/legend)** | **n/a** |
| For commercial reagents, provide supplier name, catalogue number and [RRID](https://scicrunch.org/resources), if available. | Primary Insulin & Pro-insulin antibody (for analysis of perifusion): (10R-I136a, Biosynth; also referred to as D6C4)  Secondary Insulin & Proinsulin antibody, biotinylated (for analysis of perifusion): 61R-I136b-BT, Biosynth; also referred to as D3E7)  These are referred to in citations 17 (Mitok et. al. JBC 2018) and 41 (Emfinger et. al. JCI Insight 2022). Citation 17 is referred to in the materials and methods section of the preprint, p.24 as appropriate. |  |
|  |  |  |
| **DNA and RNA sequences** | **indicate where provided: page no/section/legend)** | **n/a** |
| **Short novel DNA or RNA including primers, probes:** Sequences should be included or deposited in a public repository. |  | NA |
|  |  |  |
| **Cell materials** | **indicate where provided: page no/section/legend** | **n/a** |
| **Cell lines:** Provide species information, strain. Provide accession number in repository **OR** supplier name, catalog number, clone number, **OR** RRID. |  | NA |
| **Primary cultures:** Provide species, strain, sex of origin, genetic modification status. | Islets from laboratory mice (Mus Musculus) of both sexes were isolated. The strains used were: A/J, C57BL/6J (B6), 129S1/SvlmJ (129), NOD/ShiLtJ (NOD), NZO/HILtJ (NZO), CAST/EiJ (CAST), PWK/PhJ (PWK), and WSB/EiJ (WSB). Information about isolation procedures is indicated on p. 22 (preprint) in the Materials & Methods section. |  |
|  |  |  |
| **Experimental animals** | **indicate where provided: page no/section/legend)** | **n/a** |
| **Laboratory animals or Model organisms:** Provide species, strain, sex, age, genetic modification status. Provide accession number in repository **OR** supplier name, catalog number, clone number, **OR** RRID. | The strains used were: A/J (RRID:IMSR_JAX:000646), C57BL/6J (B6) (RRID:IMSR_JAX:000664), 129S1/SvlmJ (129) (RRID: IMSR_JAX:002448), NOD/ShiLtJ (NOD) (RRID:IMSR_JAX:001976), NZO/HILtJ (NZO) (RRID:IMSR_JAX:002105), CAST/EiJ (CAST) (RRID:IMSR_JAX:000928), PWK/PhJ (PWK) (RRID:IMSR_JAX:003715), and WSB/EiJ (WSB)( RRID:IMSR_JAX:001145). All strains except CAST and NZO were maintained in house. CAST (line 000928, RRID IMSR_JAX:000928) and NZO (line 002105 IMSR_JAX:002105) were purchased from Jackson Laboratories (Bar Harbor, ME). This is indicated on pages 7 and 23. |  |
| **Animal observed in or captured from the field:** Provide species, sex, and age where possible. |  | NA |
|  |  |  |
| **Plants and microbes** | **indicate where provided: page no/section/legend)** | **n/a** |
| **Plants:** provide species and strain, ecotype and cultivar where relevant, unique accession number if available, and source (including location for collected wild specimens). |  | NA |
| **Microbes:** provide species and strain, unique accession number if available, and source. |  | NA |
|  |  |  |
| **Human research participants** | **indicate where provided: page no/section/legend) or state if these demographics were not collected** | **n/a** |
| If collected and within the bounds of privacy constraints report on age, sex and gender or ethnicity for all study participants. |  | NA |

**Design:**

| **Study protocol** | **indicate where provided: page no/section/legend)** | **n/a** |
| --- | --- | --- |
| If study protocol has been pre-registered, provide DOI. For clinical trials, provide the trial registration number **OR** cite DOI. |  | NA |
|  |  |  |
| **Laboratory protocol** | **indicate where provided: page no/section/legend)** | **n/a** |
| Provide DOI **OR** other citation details if detailed step-by-step protocols are available. |  | NA |
|  |  |  |
| **Experimental study design (statistics details)** | | |
| **For in vivo studies:** State whether and how the following have been done | **indicate where provided: page no/section/legend. If it could have been done, but was not, write not done** | **n/a** |
| Sample size determination | The in vivo tracking data (glucose, insulin, and triglycerides) were monitored as per citation 17 (Mitok et al 2018) but were not part of the data analysis. They were used for the purposes of monitoring the health of the animals. All correlation comparisons used the previously collected data from that citation. | NA |
| Randomisation |  | NA |
| Blinding |  | NA |
| Inclusion/exclusion criteria |  | NA |
|  |  |  |
| **Sample definition and in-laboratory replication** | **indicate where provided: page no/section/legend** | **n/a** |
| State number of times the experiment was replicated in laboratory. | Stated on p.23-24 and 30 in the methods section. Between 2 and 4 animals were imaged in a single day and thus the imaging experiments required multiple days to complete. Specific dates, which specific animals were assayed, and other information for experiments is provided in the raw and processed data on Dryad (https://datadryad.org/stash/dataset/doi:10.5061/dryad.j0zpc86jc) and Zenodo (https://doi.org/10.5281/zenodo.7776230) |  |
| Define whether data describe technical or biological replicates. | Stated on p.23-24 and 30 in the methods section as well as in the legends for Figures 1, 3 and Figure 1-supplement 1. |  |
|  |  |  |
| **Ethics** | **indicate where provided: page no/section/legend** | **n/a** |
| **Studies involving human participants:** State details of authority granting ethics approval (IRB or equivalent committee(s), provide reference number for approval. |  | NA |
| **Studies involving experimental animals:** State details of authority granting ethics approval (IRB or equivalent committee(s), provide reference number for approval. | Statement of approval (protocol # A005821-R01) is in the Study Approval section on p.30 |  |
| **Studies involving specimen and field samples:** State if relevant permits obtained, provide details of authority approving study; if none were required, explain why. |  | NA |
|  |  |  |
| **Dual Use Research of Concern (DURC)** | **indicate where provided: page no/section/legend** | **n/a** |
| If study is subject to dual use research of concern regulations, state the authority granting approval and reference number for the regulatory approval. |  | NA |

**Analysis:**

| **Attrition** | **indicate where provided: page no/section/legend** | **n/a** |
| --- | --- | --- |
| Describe whether exclusion criteria were preestablished. Report if sample or data points were omitted from analysis. If yes report if this was due to attrition or intentional exclusion and provide justification. | One perifusion curve was omitted due to technical issues. This is noted in the figure 3 legend on p.40 |  |
|  |  |  |
| **Statistics** | **indicate where provided: page no/section/legend** | **n/a** |
| Describe statistical tests used and justify choice of tests. | Described on p.29 for perifusion experiments and pages 26, 27, and 30 for the calcium imaging correlations and data resource |  |
|  |  |  |
| **Data availability** | **indicate where provided: page no/section/legend** | **n/a** |
| For newly created and reused datasets, the manuscript includes a data availability statement that provides details for access or notes restrictions on access. | Data resource availability statement is p.29 |  |
| If newly created datasets are publicly available, provide accession number in repository **OR** DOI **OR** URL and licensing details where available. | New resources are described p.28-29. Repository links:  https://doi.org/10.5061/dryad.j0zpc86jc, https://zenodo.org/record/7776230, https://zenodo.org/record/7776210,  https://data-viz.it.wisc.edu/FounderCalciumStudy/, https://github.com/byandell/FounderCalciumStudy, https://rstudio.it.wisc.edu/FounderCalciumStudy |  |
| If reused data is publicly available provide accession number in repository **OR** DOI **OR** URL, **OR** citation. | Previously published proteomic and in vivo data (from citation 17, Mitok et al. 2018 Journal of Biological Chemistry DOI). |  |
|  |  |  |
| **Code availability** | **indicate where provided: page no/section/legend** | **n/a** |
| For all newly generated custom computer code/software/mathematical algorithm or re-used code essential for replicating the main findings of the study, the manuscript includes a data availability statement that provides details for access or notes restrictions. | Data resource availability statement is p.29 |  |
| If newly generated code is publicly available, provide accession number in repository, **OR** DOI **OR** URL and licensing details where available. State any restrictions on code availability or accessibility. | https://zenodo.org/record/7776230, https://zenodo.org/record/7776210,  https://data-viz.it.wisc.edu/FounderCalciumStudy/, https://github.com/byandell/FounderCalciumStudy, https://rstudio.it.wisc.edu/FounderCalciumStudy |  |
| If reused code is publicly available provide accession number in repository **OR** DOI **OR** URL, **OR** citation. | https://zenodo.org/record/7776230, https://zenodo.org/record/7776210,  https://github.com/hrfoster/Merrins-Lab-Matlab-Scripts |  |

**Reporting**

MDAR framework recommends adoption of discipline-specific guidelines, established and endorsed through community initiatives. Journals have their own policy about requiring specific guidelines and recommendations to complement MDAR.

| **Adherence to community standards** | **indicate where provided: page no/section/legend** | **n/a** |
| --- | --- | --- |
| State if relevant guidelines (e.g., ICMJE, MIBBI, ARRIVE) have been followed, and whether a checklist (e.g., CONSORT, PRISMA, ARRIVE) is provided with the manuscript. | We did not state this in the manuscript |  |
